# Discovering *in vitro* spermatogenesis stimulating factors

**DOI:** 10.1038/cddis.2015.303

**Published:** 2015-10-22

**Authors:** J Chaudhary, F K Hamra

**Affiliations:** 1 Department of Pharmacology, Cecil H. & Ida Green Center for Reproductive Biology Sciences, University of Texas Southwestern Medical Center, 6001 Forest Park Road, Dallas, TX 75390, USA

Spermatozoan development encompasses pre-meiotic, meiotic and post-meiotic cellular processes adapted to genetically diversify and then vertically transmit haploid genomes via fertilization. Defined culture systems supporting spermatogonial stem cell differentiation through meiosis will provide long-sought-after experimental platforms to study spermatogenesis, and to generate haploid gametes *in vitro*. Despite implications to advance science and medicine, germline-intrinsic signaling mechanisms sufficient to support spermatogenic cell differentiation in culture without somatic cells remain largely undiscovered. A *Cell Death and Discovery* article by Chapman *et al.*^1^ now reports spermatogenic growth factors sufficient for robust serum/soma-free rat spermatogonial differentiation. Culturing differentiating spermatogonia in a defined system opens new doors to investigate molecular mechanisms controlling pre-meiotic steps in spermatogonial development, and to discover factors that drive meiotic progression *in vitro*.

Spermatozoa are continuously produced in the testes through the developmental processes of spermatogenesis and spermiogenesis.^2^ Spermatogonial stem cells maintain spermatogenesis by their abilities to self-renew or develop into syncytia of mitotically amplifying differentiating spermatogonia that initiate meiosis as spermatocytes.^3^ Newly formed spermatocyte syncytia traverse the Sertoli cell blood–testis barrier to enter the seminiferous tubules.^2^ Once within the seminiferous epithelium's ‘adluminal' compartment, spermatocytes complete two meiotic divisions to generate nascent haploid germ cells termed round spermatids.^2^ As requisite for round spermatids to mature into fully elongated, motile spermatozoa, they must transform dramatically in size, shape and organelle composition through the post-meiotic process of spermiogenesis.^2^

Culture systems in which spermatids are robustly produced from spermatogonial stem cells hold potential to positively impact a wide portfolio of global health issues related to over population, lack of family planning and the spread of infectious disease.^4, 5^ The ability to generate fertilization-competent spermatids (i.e. gametes) from cryopreservable spermatogonial stocks will provide prospective treatments for male infertility, enable methods to conserve species and facilitate the production of novel transgenic organisms.^6^ Benefits of *in vitro* spermatogenesis are being held at bay because culture systems that effectively support spermatogonial stem cell proliferation and/or early pre-meiotic differentiation steps do not exist for most mammalian species outside rodents. In rodents, donor spermatogonial stem cells can be cultured through meiosis in recipient testes,^3^ or in organ culture within seminiferous tubules.^7^ Going forward, chemically defined, soma-free culture systems that robustly support spermatid production will need to be established from diverse species to realize the full potential of *in vitro* spermatogenesis in science, industry and medicine.

As a primary example, defined culture systems for spermatogenesis and spermiogenesis are needed to help study traits that influence sperm counts. Azoospermia is a disorder that currently renders millions of couples infertile due to reduced spermatozoa production.^8, 9, 10^ Cases of azoospermia, including spermatogenic arrest, are caused by diverse traits that impact various steps in spermatozoa development.^8, 9, 10^ Highly defined experimental systems are needed to obtain a more detailed understanding on how genetic interactions control spermatozoa development. Once established, chemically defined culture systems supporting spermatogenesis and/or spermiogenesis hold potential to help classify germline-intrinsic and -extrinsic molecular mechanisms controlling distinct steps in spermatozoa development. Detailed knowledge on how gene products control spermatozoa development will facilitate formulating therapies that boost sperm counts to treat multiple azoospermia types. Spermatids also provide fertilization-competent gametes if mechanically injected into oocytes.^11^ Defined culture systems that support healthy spermatid maturation into advanced elongating steps will enable men with compatible cases of spermatogenic arrest to parent their own children.^11^

Large knowledge gaps persist on signaling pathways and metabolic states within germ cells that can support their differentiation through pre-meiotic, meiotic or post-meiotic steps of spermatozoa development independent of a somatic environment. Because differentiating spermatogenic cells do not survive in culture without somatic cells, the ability to culture mammalian stem cells through spermatogenesis or spermiogenesis in defined systems remains a long-standing technical hurdle in science. Lack of success at culturing stem cells through spermatogenesis in defined systems has fueled hypotheses that the complexity of specific germ cell and somatic cell relationships in testes is essential for the process of spermatogenesis, which explains why highly pure mammalian spermatogonia have not developed into meiosis, much less through meiosis without somatic cells.

A similar theory on why spermatogonial stem cells have yet to be cultured through spermatogenesis without somatic cells is that key spermatogenic growth factors ‘simply' remain undiscovered. As a critical first step, polypeptides encoded by *Glial cell line derived neurotrophic factor* (*Gdnf*) and *Fibroblast growth factor 2* (*Fgf2*) are able to maintain mouse spermatogonial stem cell proliferation long-term in serum-free media without somatic cells.^12^ GDNF and FGF2 also support long-term proliferation of mouse and rat spermatogonial stem cells on fibroblast feeder layers in serum-free media.^13, 14^ Still, in contrast to highly defined culture systems that maintain spermatogonial stem cell proliferation, few molecules have been clearly established to support syncytial growth of differentiating spermatogonia from stem cells in a defined medium.

One germline receptor, ERBB3, was recently genetically annotated as essential for syncytial growth of rat differentiating spermatogenic cells in a serum/soma-free culture medium containing NRG1, GDNF, FGF2 and ATRA.^13^ Because ERBB3 is a transmembrane receptor for NRG1, Chapman *et al.* proceeded to combine pharmacological and genetic approaches to delineate a NRG1/ERBB3/ERBB2 signaling pathway in the rat germline downstream of all-trans retinoic acid that effectively supported pre-meiotic steps of spermatogenesis in a defined medium without somatic cells.^1^ However, rat *Erbb3*-null germlines fully supported spermatogenesis in recipient rat testes.^13^ Thus, spermatogenic factors that supported spermatogonial differentiation in recipient testes were either absent from or inactive in cultures of the *Erbb3*-null germlines.^13^

After ERBB-family subunits that transduced NRG1 signaling in spermatogonia were elucidated in a defined system, Chapman *et al.*^1^ tested the hypothesis that an alternate, ERBB-independent pathway could support spermatogonial development *in vitro* without somatic cells. Indeed, based on studies where testicular KIT activity was required for spermatogonial development,^15^ Chapman *et al.* demonstrated that a primary candidate, KITL, could stimulate soma-free syncytial growth of differentiating spermatogonia in the absence or presence of ERBB-family inhibitors, which otherwise phenocopied NRG1's effects on germ cell development *in vitro*.^1^ Thus, KITL acted independent of ERBB-signaling in the rat germline to support syncytial growth of differentiating spermatogenic cells without somatic cells (Figure 1).

In summary, studies by Chapman *et al.* highlight how NRG1 and KITL signal through alternate transmembrane receptors to support differentiating spermatogonia survival during clonal development *in vitro* (Figure 1). By analogy, it remains to be determined if identifying growth factor receptors that act directly in spermatocytes will facilitate the discovery of additional spermatogenic factors that cooperate with NRGs, KITL and retinoic acid to promote meiotic progression from differentiating spermatogonia in the culture dish. Despite intricate germ cell an somatic cell associations comprising mammalian seminiferous epithelia,^2^ Chapman *et al.* demonstrate that it remains possible to molecularly dissect actions of spermatogenic growth factors in the ‘test tube' during differentiation from primary stem cell lines. The ability to conditionally stimulate pre-meiotic development from stem cell lines in chemically defined cultures builds credence for the concept that systematic addition of factors to differentiating spermatogonial clones without somatic cells may some day lead to the derivation of haploid gametes *in vitro*.

## Figures and Tables

**Figure 1:**
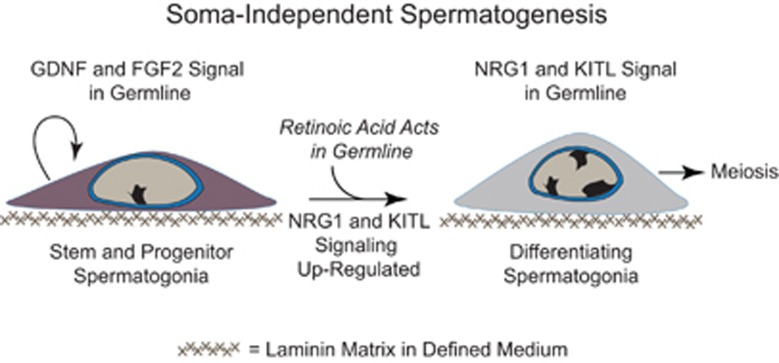
In a new issue of *Cell Death and Discovery*, Chapman *et al.* report alternate growth factor pathways activated by NRG1 and KITL that are necessary for retinoic acid-induced syncytial growth of rat spermatozoan progenitors *in vitro*.^1^ As illustrated, FGF2 and GDNF maintain undifferentiated spermatogonial stem and progenitor cells in culture on laminin. Retinoic acid acts in the germline to drive transformation of undifferentiated spermatogonia into nascent differentiating spermatogonia. Polypeptide growth factors NRG1 and KITL are required for survival of differentiating spermatogonia on laminin without somatic cells. The defined cultures provide long-sought-after, soma-free platforms to study spermatogonial differentiation, and to discover additional spermatogenic factors for stimulating spermatogonia to undergo meiosis
